# Prevalence and Factors Associated with Herpes Simplex Virus Type 2 Infection in Patients Attending a Baltimore City Emergency Department

**DOI:** 10.1371/journal.pone.0102422

**Published:** 2014-07-18

**Authors:** Eshan U. Patel, Melanie A. Frank, Yu-Hsiang Hsieh, Richard E. Rothman, Amy E. O. Baker, Chadd K. Kraus, Judy Shahan, Charlotte A. Gaydos, Gabor D. Kelen, Thomas C. Quinn, Oliver Laeyendecker

**Affiliations:** 1 Division of Intramural Research, NIAID, NIH, Baltimore, Maryland, United States of America; 2 Department of Medicine, Johns Hopkins University, Baltimore, Maryland, United States of America; Cincinnati Childrens Hospital Medical Center, United States of America

## Abstract

**Objectives:**

Herpes simplex virus type 2 (HSV-2) is a common sexually transmitted disease, but there is limited data on its epidemiology among urban populations. The urban Emergency Department (ED) is a potential venue for surveillance as it predominantly serves an inner city minority population. We evaluate the seroprevalence and factors associated with HSV-2 infection among patients attending the Johns Hopkins Hospital Adult Emergency Department (JHH ED).

**Methods:**

An identity unlinked-serosurvey was conducted between 6/2007 and 9/2007 in the JHH ED; sera were tested by the Focus HerpeSelect ELISA. Prevalence risk ratios (PRR) were used to determine factors associated with HSV-2 infection.

**Results:**

Of 3,408 serum samples, 1,853 (54.4%) were seropositive for HSV-2. Females (adjPRR  = 1.47, 95% CI 1.38–1.56), non-Hispanic blacks (adjPRR  = 2.03, 95% CI 1.82–2.27), single (adjPRR  = 1.15, 95% CI 1.07–1.25), divorced (adjPRR  = 1.28, 95% CI 1.15–1.41), and unemployed patients (adjPRR  = 1.13, 95% CI 1.05–1.21) had significantly higher rates of HSV-2 infection. Though certain zip codes had significantly higher seroprevalence of HSV-2, this effect was completely attenuated when controlling for age and gender.

**Conclusions:**

Seroprevalence of HSV-2 in the JHH ED was higher than U.S. national estimates; however, factors associated with HSV-2 infection were similar. The high seroprevalence of HSV-2 in this urban ED highlights the need for targeted testing and treatment. Cross-sectional serosurveys in the urban ED may help to examine the epidemiology of HSV-2.

## Introduction

Herpes simplex virus type 2 (HSV-2) is one of the most common sexually transmitted infections worldwide. HSV-2 is primarily characterized by painful genital lesions and is a major cause of genital ulcer disease [1]. Observational studies suggest HSV-2 is associated with a three-fold increase in human immunodeficiency virus (HIV) acquisition [2]. HSV-2 can easily be transmitted by close contact between sexual partners. Although perinatal transmission is less common, it can be deadly to the fetus if it occurs [3], [4]. HSV-2 infection is often subclinical [5], and previous studies have shown that up to 87.4% of HSV-2 infected individuals in the United States are unaware of their infection [6], [7]. Thus, HSV-2 prevalence can only be monitored by serological surveillance.

The National Health and Nutrition Examination Survey (NHANES) has been the major serological study for the general population. In 2005–2010, NHANES reported that the seroprevalence of HSV-2 in the United States was 15.7% [8]. Modeled after NHANES, the 2004 New York City (NYC) Health and Nutrition Examination Survey (HANES) reported a higher HSV-2 seroprevalence (27.9%) [9]. Both studies indicated that being female, non-Hispanic black, and socioeconomically disadvantaged were factors associated with HSV-2 infection [6], [8], [9]. Aside from NYC HANES, recent data on HSV-2 seroprevalence and associated factors in urban settings is limited.

The urban Emergency Department (ED) is a potential venue for surveillance of HSV-2. The Johns Hopkins Hospital Adult Emergency Department (JHH ED) primarily sees a high proportion of individuals from the surrounding predominantly minority community, many of whom have poor access to healthcare and often use the ED for primary healthcare [10]–[12]. This patient population can be used to establish specific demographics associated with HSV-2 infection in disadvantaged urban settings. Urban ED surveillance has previously been beneficial in monitoring the prevalence of sexually transmitted infections such as HIV, chlamydia, and gonorrhea [10], [11], [13], [14]. Including HSV-2 serology in this well-defined surveillance method may help to characterize its epidemiology among urban populations. The purpose of this study was to describe the seroprevalence and associated factors of HSV-2 infection in the JHH ED in Baltimore City, Maryland.

## Methods

### Ethics Statement

The study was conducted according to the ethical standards set forth by the institutional review board of the Johns Hopkins University and the Helsinki Declaration of the World Medical Association. As the study was an identity unlinked serosurvey, the need to obtain consent was waived. No participants were recruited or followed-up during the course of this study. All laboratory testing was performed on the remnants of blood samples obtained for patient care.

### Study Population and Laboratory Testing

An identity-unlinked serosurvey was conducted from 6/2007 to 9/2007 in the JHH ED in Baltimore City, Maryland. The protocol for the study was the same as previously described [10]. In brief, eligible patients were ≥18 years of age, required blood drawn for a medical reason, and had matched chart review data. For patients who attended the JHH ED multiple times within the study period, only one serum sample was included for testing. Patients who listed their home as a non-US location were excluded from the study population. Serological HSV-2 infection status was determined by the Focus HerpeSelect-2 enzyme-linked immunosorbent assay (Focus Technologies, Cypress, California, U.S.) per kit protocol.

### Statistical Analysis

Zip code boundary data from 2007 was obtained from the Maryland Department of Planning, and only zip codes with ≥40 patients were included in the analyses. For HSV-2 seroprevalence, exact 95% confidence intervals (CIs) were assessed from a binomial distribution. Prevalence risk ratios (PRRs) of HSV-2 seroprevalence were estimated using a Poisson regression model. Adjusted PRRs (adjPRRs) were estimated using a multivariate regression model for all factors shown to have an association (*p*<0.1) in the univariate analysis. In all statistical analyses, *p*<0.05 determined significant differences. Data analyses for the study were performed in STATA V.11.2 (StataCorp, College Station, Texas, U.S.).

## Results

### Study Population

The study population included 3,408 unique samples (median age  = 45.3 years, interquartile range 33.2–56.8). The majority of samples came from females (54.0%), non-Hispanic blacks (67.4%), the unemployed (57.8%), unmarried individuals (75.6%), and Baltimore City residents (78.5%). Almost one-third of the patients sampled did not have any form of health insurance (32.0%). All results are available in Table S1.

### Seroprevalence of HSV-2

In the study population, 1,853 (54.4%) were seropositive for HSV-2. Seroprevalence was highest among females (63.6%), patients older than 25 years of age (57.9%), non-Hispanic blacks (66.3%), divorced (66.3%) or widowed (66.2%) patients, the unemployed (58.3%), patients on Medicare (55.5%), and Baltimore City residents (59.4%) (Table 1).

**Table 1:** Factors associated with HSV-2 infection in the JHH ED (n = 3,408).

	n	Prevalence (95% CI)	PRR (95% CI)	adjPRR (95% CI)^1^
Gender				
Male	1,568	43.5% (41.0–46.0)	1.00	1.00
Female	1,840	63.6% (61.4–65.8)	1.46 (1.37–1.56)*	1.47 (1.38–1.56)*
Age				
18–24	386	26.4% (22.1–31.1)	1.00	1.00
25–34	560	49.3% (45.1–53.5)	1.87 (1.55–2.25)*	2.07 (1.73–2.48)*
35–44	729	62.1% (58.5–65.7)	2.35 (1.97–2.80)*	2.57 (2.16–3.05)*
45–54	769	61.9% (58.4–65.3)	2.34 (1.97–2.79)*	2.55 (2.15–3.03)*
55–64	467	59.3% (54.7–63.8)	2.24 (1.87–2.69)*	2.53 (2.11–3.02)*
>65	497	54.1% (49.6–58.6)	2.05 (1.70–2.46)*	2.30 (1.90–2.80)*
Race				
Non-Hispanic whites	918	29.1% (26.2–32.1)	1.00	1.00
Non-Hispanic blacks	2,297	66.3% (64.3–68.2)	2.28 (2.05–2.53)*	2.03 (1.82–2.27)*
Other	193	33.2% (26.6–40.3)	1.14 (0.91–1.43)	1.20 (0.96–1.50)
Marital Status				
Married	813	45.5% (42.0–49.0)	1.00	1.00
Single	2,086	55.1% (52.9–57.2)	1.21 (1.11–1.32)*	1.15 (1.07–1.25)*
Divorced	297	66.3% (60.6–71.7)	1.46 (1.30–1.63)*	1.28 (1.15–1.41)*
Widowed	195	66.2% (59.0–72.8)	1.45 (1.28–1.65)*	1.13 (1.01–1.27)†
Employment Status				
Employed	910	47.1% (43.9–50.4)	1.00	1.00
Unemployed	1,969	58.3% (56.0–60.4)	1.24 (1.14–1.34)*	1.13 (1.05–1.21)*
Retired	295	54.6% (48.7–60.4)	1.16 (1.02–1.31)†	1.15 (1.00–1.32)†
Other	144	55.6% (47.1–63.8)	1.18 (1.00–1.39)	1.13 (0.97–1.31)
Insurance				
Insured	1,624	53.7% (51.2–56.1)	1.00	-
Uninsured	1,092	54.9% (51.8–57.8)	1.02 (0.95–1.10)	-
Medicare	647	55.5% (51.6–59.4)	1.03 (0.95–1.12)	-
Residence				
Baltimore City	2,675	59.4% (57.6–61.3)	1.00	1.00
Other Maryland	549	36.8% (32.7–41.0)	0.62 (0.55–0.69)*	0.85 (0.77–0.95)*
Other State	184	33.2% (26.4–40.5)	0.56 (0.45–0.69)*	0.83 (0.67–1.03)
**Overall**	3,408	54.4% (52.7–56.1)	-	-

*p<0.01.

† *p*<0.05.

1 Adjusted for gender, age, race, marital status, employment status, and residence.

**Figure 1:**
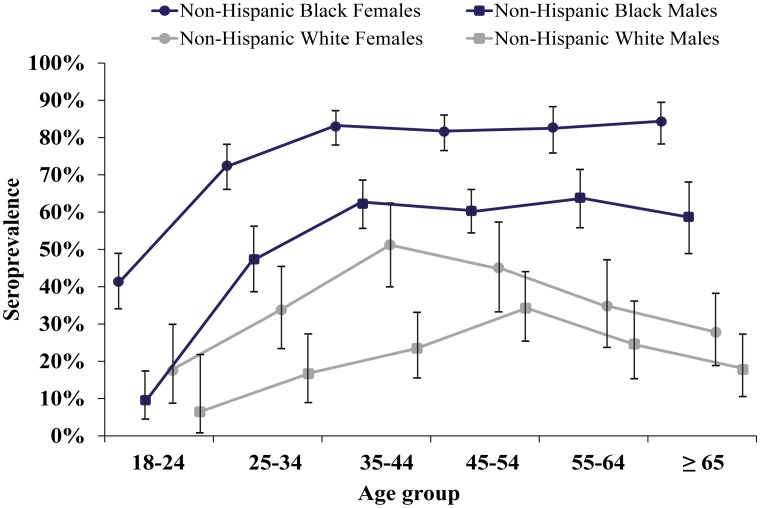
HSV-2 seroprevalence in the JHH ED stratified by age, gender, and race. Data are shown for 3,215 non-Hispanic black and non-Hispanic white patients that attended the JHH Adult ED in Baltimore City. Blue lines indicate non-Hispanic blacks and grey lines indicate non-Hispanic whites. Circles and squares denote females and males, respectively. Bars display 95% confidence intervals.

The highest seroprevalence of HSV-2 was seen in non-Hispanic black females (Figure 1). Those 18–24 years of age had a seroprevalence of 41.3% (74/179). Seroprevalence plateaued in non-Hispanic black females ≥35 years of age at 82.8% (736/889). Among non-Hispanic black males, the seroprevalence was at least 20% less than that of the non-Hispanic black females for all age groups, plateauing for patients ≥35 years of age at 61.4% (477/777). The seroprevalence of non-Hispanic white females increased to a maximum of 49.3% (35/71) among patients 35–44 years of age, followed by a significant decrease in seroprevalence of 35.2% (81/230) in those ≥45 years of age. Non-Hispanic white males followed the same trend as non-Hispanic white females, but peaked at 33.7% (34/101) among patients 45–54 years of age, with a significant decrease in seroprevalence of 20.9% (34/163) in those ≥55 years of age.

**Figure 2:**
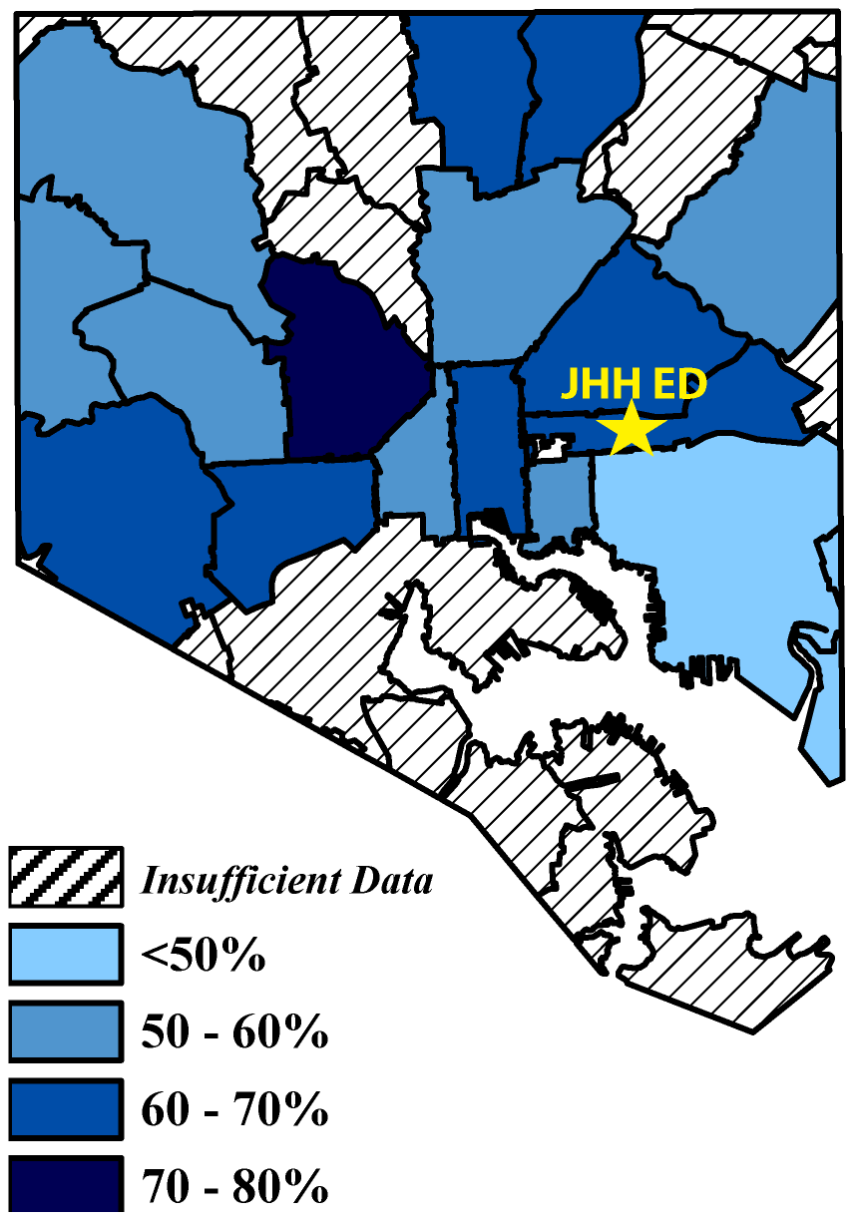
HSV-2 seroprevalence in the JHH ED by Baltimore City zip code. Only zip codes that had ≥40 patient visits to the JHH ED are included in the map as a function of HSV-2 seroprevelance. The map was constructed using 2007 zip code boundary data obtained from the Maryland Department of Planning. The star indicates the JHH ED in zipcode 21205.

The seroprevalence of HSV-2 varied significantly by zip code. When identified as residing within Baltimore City, the seroprevalence ranged from a high of 73.9% (85/115, 95% CI 64.9%–81.7%) in zip code 21217 to a low of 45.3% (101/223, 95% CI 38.6%–52.1%) in zip code 21224 (Figure 2). The seroprevalence of HSV-2 was lower for patients who were non-Maryland residents (32.6%, 61/187, 95% CI 26.0%–40.0%). Relative to the zip code where the JHH ED is located (21205), two zip codes (21224 and 21222), and patients who lived outside of Maryland had significantly lower seroprevalence of HSV-2. However, the effect was completely attenuated after controlling for age and gender, apart from those living outside of Maryland.

### Factors Associated with HSV-2 Infection

In the univariate analyses, gender, age, race, employment status, marital status, and residency were significantly associated with HSV-2 prevalence (Table 1). All associated factors remained stable in predicting risk in the multivariate model. Females (adjPRR  = 1.47, 95% CI 1.38–1.56), non-Hispanic blacks (adjPRR  = 2.03, 95% CI 1.82–2.27), single (adjPRR  = 1.15, 95% CI 1.07–1.25), divorced (adjPRR  = 1.28, 95% CI 1.15–1.41), and unemployed patients (adjPRR  = 1.13, 95% CI 1.05–1.21) were at a significantly higher risk for HSV-2 infection. There was no association between insurance status and HSV-2 seroprevalence (Table 1).

## Discussion

This is the first reported study that examines seroprevalence and associated risk factors of HSV-2 in patients attending an inner city ED. The results of this study indicate a high seroprevalence (54.4%) of HSV-2 in the JHH ED, a likely reflection of Baltimore City ED patients. The findings of higher seroprevalence among females, non-Hispanic blacks, patients ≥25 years of age, unmarried, and unemployed patients are similar to previous studies in urban and general populations [8], [9], [15]–[17].

Due to the chronic nature of HSV-2 infection and results of previous studies, it was expected that older patients would have the highest seroprevalence [8]. The largest difference in seroprevalence among age groups was observed between those 18–24 years of age and those 25–34 years of age. Between every age group ≥35 years, overall seroprevalence did not differ. These results may be explained by an increase in HSV-2 incidence in the 20 s age group and a decrease in HSV-2 incidence in the mid-30 s age, as previously reported from a population-based birth cohort study [15]. Nevertheless, the high seroprevalence seen across all ages suggests the need for HSV-2 prevention education to decrease acquisition and transmission.

Although a disparity in HSV-2 seroprevalence between non-Hispanic blacks and non-Hispanic whites is well established [6], [18], the high prevalence among non-Hispanic blacks in the current study is concerning. In older aged non-Hispanic whites, there was a decrease in HSV-2 prevalence, but in non-Hispanic blacks, high seroprevalence persisted (see Figure 1). Most alarming is that 82.8% of non-Hispanic black females ≥35 years of age were seropositive. Inner city non-Hispanic black females are significantly at-risk for HSV-2 infection and would certainly benefit from an HSV-2 vaccine.

The findings from this study support previous studies that suggested a higher seroprevalence in urban settings compared to the general population [9], [16], [17]. Not only is the overall seroprevalence in this study population higher than national estimates from NHANES [8], it is higher than the overall seroprevalence reported in New York City [9] and among military recruits [17]. The majority of patients that used the JHH ED were non-Hispanic blacks, which may explain the higher overall prevalence than previous studies. However, even the seroprevalence among the non-Hispanic black female population and non-Hispanic black male population in this study is respectively higher than the seroprevalence found in NYC HANES, NHANES and military recruits [6], [9], [17]. While surveillance of the general population is necessary, continued surveillance of high-risk populations is also important as results greatly differ.

Increasing the diagnosis of HSV-2 in the urban ED may help to expand care, prevent future outbreaks in seropositive patients, and subsequently reduce transmission [19]–[22]. Although the current guidelines from the U.S. Preventative Services Task Force recommend against routine serologic screening for HSV-2 [23], an algorithm for targeted HSV-2 testing may benefit high-risk populations. This study highlights certain factors associated with HSV-2 infection in an urban ED, but future studies should evaluate additional risk factors that have not been examined in this setting. For instance, substance abuse and sexual risk, including sexual orientation, age of sexual debut, and number of lifetime partners are key risk factors for HSV-2 infection in the general population [7], [8], [24].

This study has limitations. As with any serosurvey, errors of misclassification in serological testing are possible. Multiple studies have demonstrated the Focus HerpeSelect-2 assay to have 96–100% sensitivity and 97–100% specificity compared to HSV-2 western blotting, and this assay is currently used for HSV-2 research diagnostics [25]. However, in men attending STD clinics in Baltimore, Maryland the assay only had 82.6% sensitivity and 97.1% specificity, which suggests that our study may contain false-negative results [26]. Additionally, the prevalence among the youngest (<20 years of age) non-Hispanic black females and males were 21% (9/43) and 5% (2/44), respectively. This provides assurance that the results seen in the current study are not due to a high false positive rate of the assay. Also, only patients who had their blood drawn were included in the study population, thus these data may not be generalizable to the entire ED patient population. However, a previous JHH ED serosurvey reported that the ED patient population without blood drawn was demographically similar to the study population [10]. Finally, due to the cross-sectional nature of this study, temporal changes in HSV-2 epidemiology cannot be assessed without future studies of the same design.

Overall, the JHH ED has the highest seroprevalence of HSV-2 ever reported from a single unselected population screening site in the U.S. Although differences in seroprevalence existed across groups, this study highlights a high seroprevalence of HSV-2 across all demographics. Enhanced public health interventions to prevent and control HSV-2 infection in high prevalence populations like this ED are severely needed. The current study also demonstrates that the ED may be a key setting to monitor the epidemiology of HSV-2.

## Supporting Information

Table S1 **Data used in analysis.** Variables coded as follows: ID, subject identifier; Insured_0Yes_1No_2Medicare, 0 =  insured 1 =  not insured 2 =  Medicare; Baltimore_Resident, 1 =  yes 2 =  no; MD_Resident 1 =  yes 2 =  no; ZipCode, resident zip code for subjects where the frequency was more than 40 individuals from that area; Gender_0F_1M, 0 =  female 1 = male; Age, age in years; Race_0W_1B_2Other, 0 = non-Hispanic white 1 = non-Hispanic black 2 = other; Marital _ Status _0 Married_1Single_2 Divorced_3 Widowed, 0 = married 1 = not married 2 = divorced 3 = widowed; Employed_ 0Yes_1No_2 Retired_3Other, 0 = employed 1 = not employed 2 = retired 3 = other; HSV2_EIA_results, 0 = HSV-2 serologically negative 1 = HSV-2 serologically positive.
(XLSX)
